# Epithelioma of Face

**Published:** 1876-12

**Authors:** 


### ARTICLE VIII.

### *Epithelioma of the Face.*

At a late meeting (Oct. 11) of the New York Pathological Society, the proceedings of which appear in the *Medical Record*, Dr. Post presented specimens of epithelioma of the right side of the face. The disease first showed itself two years ago, and gradually extended forward over the ramus of the jaw, down as low as the angle of the jaw, an l backward towards the mastoid region, involving the lobe of the ear and a portion of the meatus auditorius. There were no secondary enlargements discoverable. In the operation of extirpation of the mass, the lobe of the ear was sacrificed, and a portion of the wall of the meatus. After dissecting around the tumor and seperating it from the subjacent parts portions of the masseter muscles were removed. Cutting behind the ramus, a large vessel was encountered, which proved to be the external carotid artery. Dr. Hinton, who assisted at the operation, placed his finger upon the vessel until a ligature was applied. A formal dissection was then made, and no important blood-vessels having been cut, not much blood was lost. The tumor adhered to the parotid, but was not originally connected with it. Although a por tion of the facial nerve was removed the eye remained closed, at least up to the time that Dr. Post left her, which was in the progress of recovery from the effects of the ether. By economizing the integument around the loble of the ear and sliding the skin from the neck, a very decent covering was made for the cavity left after the operation.

